# Accurate discharge summary generation using fine tuned large language models with self evaluation

**DOI:** 10.1038/s41598-026-35552-z

**Published:** 2026-01-17

**Authors:** Wenbin Li, Hui Feng, Chao Hu, Minpeng Xu, Longlong Cheng

**Affiliations:** 1 Academy of Medical Engineering and Translational Medicine, Tianjin University, Tianjin, China; 2 China Electronics Corporation, Shenzhen, China; 3 Renmin Hospital of Wuhan University, Wuhan, China; 4 China Electronics Cloud Technology Co., Ltd., Wuhan, China; 5 China Electronics Cloud Brain (Tianjin) Technology Co., Ltd., Tianjin, China

**Keywords:** Natural Language Processing, Large Language Models, Discharge Summaries, Computational biology and bioinformatics, Health care, Mathematics and computing

## Abstract

Discharge summaries are critical for patient care continuity, clinical decision-making, and legal documentation, yet their creation is labor-intensive. Clinicians must manually integrate diverse data from multiple sources under time constraints, often leading to delays, inconsistencies, and potential omissions. This study introduces a novel framework to automate discharge summary generation using advanced natural language processing (NLP) techniques, aiming to reduce clinician workload while ensuring accurate, complete, and standardized documentation. We combine the Decomposed Low-Rank Adaptation (DoRA) fine-tuning method with a novel self-evaluation mechanism to enhance large language models (LLMs) for medical text generation. DoRA efficiently adapts pre-trained LLMs to the specialized medical domain, demonstrating superior performance over traditional methods such as LoRA and QLoRA, with a enhancement in BERTScore and a reduction in Perplexity across all evaluated models. The self-evaluation mechanism, inspired by cognitive psychology, iteratively re-feeds generated summaries together with segmented clinical data into the model, allowing it to systematically detect and correct omissions in each data segment, thereby ensuring the outputs accurately and comprehensively represent the original input. This approach was rigorously compared against few-shot prompting and Chain of Thought (CoT) methods. Extensive experiments show that self-evaluation improves BERTScore by 6.9% and 4.1% and increases ROUGE-L by 69.6% and 0.4% relative to few-shot and CoT baselines, respectively, while qualitative metrics also demonstrate consistent gains in accuracy and completeness. Our results demonstrate substantial enhancements in the quality and consistency of generated discharge summaries while reducing the time required for their creation. This research underscores the potential of AI-driven tools in healthcare documentation, reducing the time required for generating discharge summaries while improving their quality and consistency. The findings indicate promising prospects for automating medical documentation that adheres to high standards of accuracy and relevance.

## Introduction

Discharge summaries play a critical role in healthcare by providing essential information to both healthcare providers and patients^1,2^. For clinicians, these summaries offer a comprehensive overview of the patient’s hospitalization, supporting continuity of care and facilitating future consultations. For patients and their families, discharge summaries serve as valuable documents that clarify the medical care received and outline post-discharge instructions. Under the pressures of clinical workload, discharge summaries are often deprioritized, which can result in delays in patient discharge or incomplete documentation^3^. Consequently, time constraints frequently lead to summaries that lack sufficient detail and depth, potentially compromising the quality of the medical record^4^.

In fast-paced clinical environments, key details or changes in a patient’s condition maybe inadvertently omitted, undermining the completeness of the medical documentation. Additionally, the absence of standardized formats and guidelines often results in inconsistencies in how medical information is organized, making it challenging for readers to quickly extract critical insights^4^. The creation of discharge summaries requires experienced clinicians to manually integrate diverse data sources from the Hospital Information System (HIS), including test results, medical orders, pathology reports, and other relevant information, which further complicates the process.

Recent advancements in Artificial Intelligence (AI) have rapidly transitioned from theoretical research to practical applications in daily life. Large language models (LLMs), crucial in natural language processing, have shown particular promise in the medical field^5^. Their capacity for human-like conversational interactions^6,7^ has led to successful applications in paper reviewing^8^, lab result interpretation^9^, and patient data anonymization^10^. However, the implementation of LLMs in healthcare faces challenges, primarily due to the disconnect between their training on general linguistic data and the specialized terminology of medicine.

While fine-tuning methods like Low Rank Adaptation (LoRA)^11^ have been explored to bridge this gap, they have shown limited effectiveness in adapting to the intricacies of medical texts. Our study addresses these limitations by employing the DoRA^12^ fine-tuning method, which better aligns the model with medical- specific terminology and improves fine-tuning efficiency. Furthermore, we introduce a novel self-evaluation mechanism, a pioneering approach in this domain that enables the model to iteratively assess and enhance its output, improving the quality and consistency of generated discharge summaries.

This research presents an innovative end-to-end workflow for automating the creation of discharge notes from unstructured breast and thyroid surgery Hospital Information System (HIS) data. By leveraging fine-tuned LLMs and our unique self-evaluation mechanism, we have developed a system that not only reduces the time required by clinical workers but also surpasses existing methods in accuracy and efficiency. Our approach has received endorsement from healthcare professionals for its clinical relevance and utility.

This advancement represents a substantial step forward in integrating AI-driven tools within patient care settings, presenting a proof-of-concept framework demonstrating the feasibility of AI-assisted discharge summary generation within a narrowly defined clinical domain. By addressing the challenges of applying LLMs to specialized medical contexts and introducing a self-improving mechanism, our work lays the foundation for more accurate, efficient, and context-aware AI applications in healthcare.

## Methodology

### Dataset

This study utilizes a dataset derived from the Thyroid Surgery Department at a major tertiary hospital in China. The dataset comprises information from 6214 patients, retrospectively collected from January 1, 2018, to December 31, 2022. The data were accessed and extracted from the hospital’s information system between March 15, 2023, and May 20, 2023 for research purposes. These data are characterized by their fully unstructured and complex nature, presenting notable challenges for analysis and processing. Data collection and processing were conducted under strict adherence to privacy protocols within the hospital’s intranet infrastructure. This approach ensures the preservation of patient confidentiality, with robust measures in place to prevent any potential leakage of sensitive information. The use of this dataset was approved by the Medical Research Ethics Committee of Renmin Hospital of Wuhan University (Ethics Approval Number: WDRY2024-K038), under the research protocol titled ‘Integrated Multi-Omics and Artificial Intelligence Analysis in Thyroid Nodule Patients: Prediction, Subtype Identification, and Exploration of Recurrence Factors’. The Clinical Research Ethics Committee of Renmin Hospital of Wuhan University approved the conduct of the clinical study in accordance with the research protocol and the waiver of informed consent application.

**Fig. 1:**
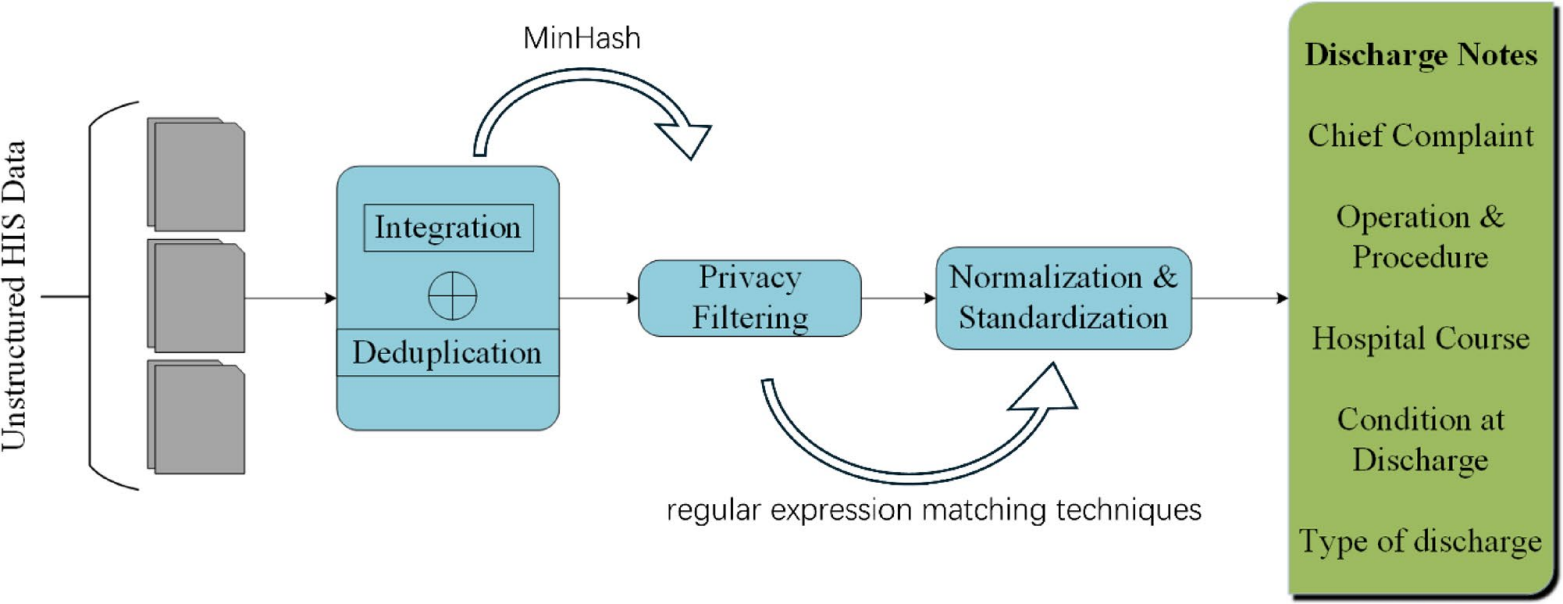
Workflow for processing unstructured Hospital Information System (HIS) data into structured discharge summaries.

As illustrated in Fig. 1, the process of transforming unstructured data from the HIS into structured discharge summaries involves several critical steps. These steps include integration, deduplication, privacy filering, and normalization. Data within the HIS are distributed across multiple tables, such as the Doctor’s Advice Information Table, Clinical Lab Information Table, Nursing Information Table, Physical Signs Information Table, Blood Glucose Information Table, Inspection Information Table, Diagnosis Table, Pathology Information Table, and Progress Notes Table. Given the interrelated nature of these data sources and the presence of duplicative entries, deduplication is a necessary step to ensure data consistency and integrity.

We employ a MinHash algorithm for efficient deduplication, effectively identifying and removing redundant data. Privacy concerns are addressed through the use of regular expression matching techniques, which filter out sensitive information from the data sets. Subsequent steps involve preparing the textual data for analysis, which includes spelling correction, acronym expansion, and the standardization of medical terminology. These pre-processing tasks are essential for enhancing the quality and usability of the data, ultimately facilitating the summarization task performed by LLMs.

During the integration process, records with identical timestamps but conflicting values (e.g., laboratory test results) were retained with provenance labels to preserve traceability. Conflicts were flagged rather than automatically resolved to avoid altering clinical evidence. This design enables downstream self-evaluation modules or human reviewers to identify and interpret potential discrepancies. Although the current model focuses on aligning generated summaries with verified input data, future work will explore automated clinical anomaly detection and resolution strategies.

The dataset spans a five-year period, comprising 6214 hospitalization episodes. Each reference discharge summary corresponds to one complete hospital stay, with hospitalization durations ranging from 2 to 28 days. The input text exhibits considerable heterogeneity in structure and length: after preprocessing, individual discharge records ranged from 636 to 2357 tokens, encompassing narratives based on multi-source HIS information including diagnostic reports, lab results, nursing notes, and physician orders. This variability ensures that the model is exposed to diverse linguistic and contextual patterns during fine-tuning, despite all cases originating from a single institution.

To ensure generalization and reliable performance evaluation, the dataset was randomly divided into training, validation, and test subsets using a 70%, 15%, and 15% split, resulting in 4349, 932, and 933 samples, respectively. Token-length statistics were computed using the same tokenizer applied during model training to characterize linguistic complexity. Both the model input sequences and the reference discharge summaries exhibited natural variability without any manual truncation or standardization, preserving authentic clinical documentation characteristics.

The reference discharge summaries used in this study were extracted directly from the HIS and authored by attending physicians or senior residents during routine clinical care. These clinicians accessed the same structured and unstructured HIS information sources (e.g., laboratory tests, pathology reports, medication orders, surgical notes, progress notes, and nursing records) that were provided to the model as input, ensuring that both human-written and model-generated summaries relied on equivalent information availability. This setting supports a fair and clinically meaningful comparison between human and model performance.

No content-based or length-based filtering, truncation, or normalization was applied to either the input texts or the reference discharge summaries. Records were excluded only when a discharge summary was severely incomplete (missing more than 50% of essential content), corrupted, or duplicated. For qualitative evaluation, a stratified random sampling strategy was employed, selecting 200 discharge summaries from the test set and categorizing them into short, medium, and long groups based on tertile-level token length to ensure representation across diverse text types.

### Model

Recent advancements in language models have shown substantial potential for medical applications. Mistral^13^, as discussed in^14^, has been particularly noted for its effectiveness in generating discharge summaries^14^. Similarly, Llama 3 has demonstrated robust performance across a variety of LLM benchmarks^15^. Due to our focus on Chinese medical data and the limited availability of Chinese corpus in previously used models, we also consider incorporating Qwen2^16,17^ to better address linguistic and regional data specifics.

### Parameter efficient fine tuning (PEFT)

The generation of discharge summaries typically adheres to a strict output format, necessitating fine-tuning of the LLM to produce standardized summaries. However, strict data protection regulations within hospital environments often restrict the ability to conduct extensive model training on external systems. Consequently, hospitals typically lack the necessary computational resources, such as GPUs, to perform full-parameter fine-tuning of large language models. This limitation underscores the importance of Parameter Efficient Fine Tuning (PEFT) in these settings. Previous studies have extensively discussed methods such as LoRA and QLoRA, which utilize the low-rank factorization of weight matrices:

$${\mathrm{W}}_{0} = {\mathrm{W}}_{0} + {\mathrm{B}} * {\mathrm{A}}^{{\mathrm{T}}}$$

However, these LoRA-based methods possess inherent drawbacks. For example, they tend to increase the general magnitude of the weight matrix components differently compared to traditional full-parameter fine-tuning^11^. This discrepancy can lead to suboptimal fine-tuning outcomes, particularly in fields like medicine, where precise terminology is crucial.

To address these issues, we employ the Weight-Decomposed Low-Rank Adaptation method, which decomposes weights into direction and magnitude components. This approach more closely approximates full-parameter fine-tuning, enhancing the model’s ability to accurately handle specialized medical terminology and context.

### Self-evaluation mechanism

In cognitive psychology, rapid, intuitive judgments are often likened to Daniel Kahneman’s 140 ‘System 1 thinking,’ which operates subconsciously. In contrast, tasks that require deliberate and methodical thought, such as mathematical calculations and logical reasoning, are categorized as ‘System 2 thinking’. Similarly, the output of Large Language Models (LLMs), especially in tasks like generating discharge summaries, can be initially rapid and intuitive, mirroring ‘System 1 thinking’. However, ensuring the reliability and completeness of these outputs often necessitates a more deliberate review process, akin to ‘System 2 thinking’.

**Fig. 2:**
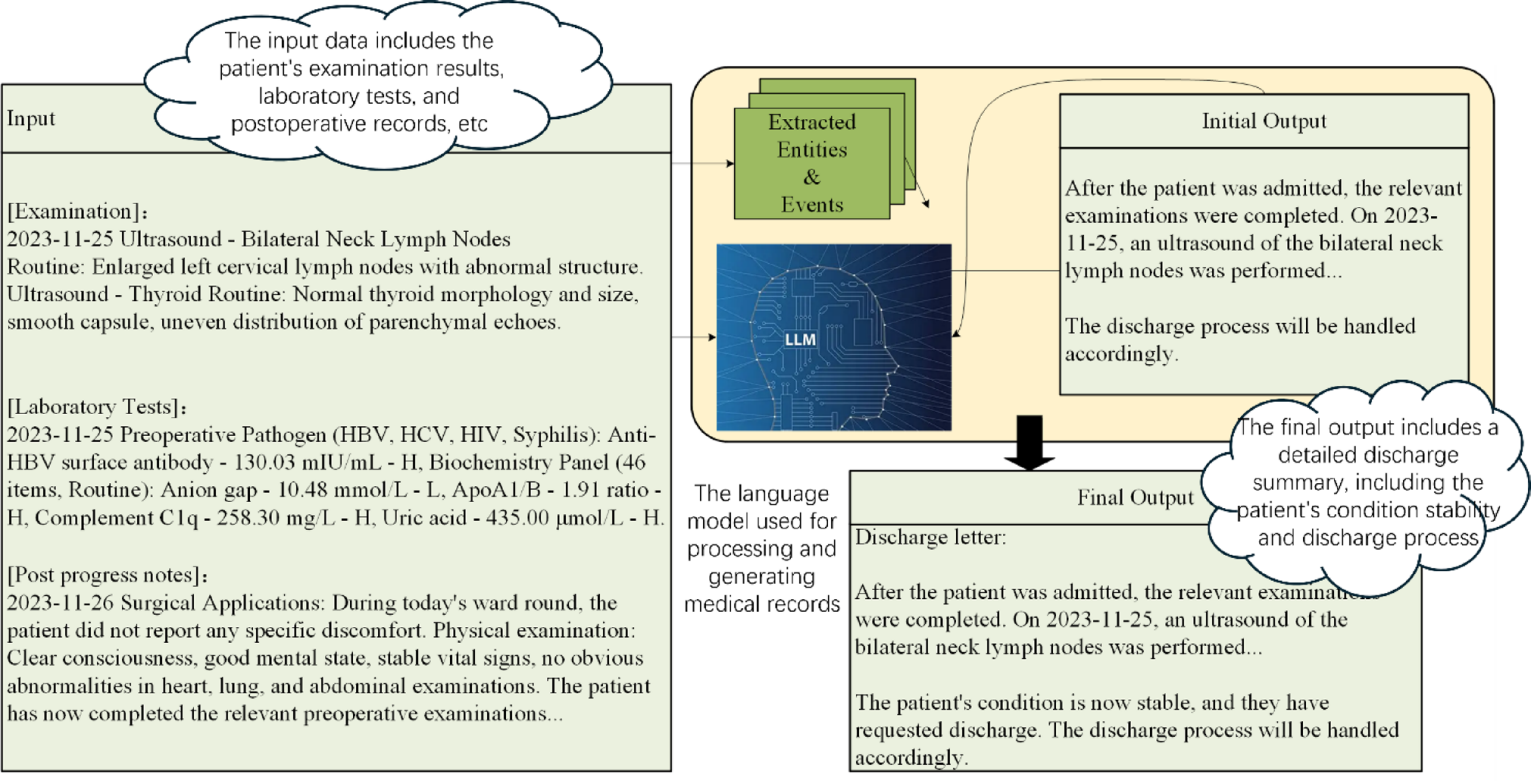
Schematic of the iterative self-evaluation mechanism for discharge summary refinement.

Despite this, our research demonstrates that LLMs are capable of self-evaluating their outputs to identify and correct omissions. This capability is facilitated by our novel self-evaluation mechanism, the general procedure of which is depicted in Fig. 2. Initially, the LLM processes the diverse information extracted from HIS data to generate a preliminary discharge summary. Concurrently, information segmentation tools decompose the original input into distinct entities and events.

Upon generating the initial summary, this output, along with segments of the original data, is re-fed into the LLM. This process allows the model to determine whether it can locate the original data’s details within the discharge summary. If details are found to be missing, the model identifies this as an omission and adjusts the summary accordingly. This iterative process continues for each data segment. Finally, the most recent summary and the complete original input are reintegrated into the LLM to assess if the enhanced summary satisfactorily represents the final, accurate discharge report. Our proposed self-evaluation mechanism operates as a model-internal process. To prevent infinite loops and ensure computational efficiency, we implemented two stopping criteria: (1) a maximum of N iteration cycles (empirically set to 3 based on preliminary experiments showing diminishing returns beyond this point), and (2) an early termination if the model’s final evaluation deems the summary “complete” for the entire input X. The LLM re-ingests its own generated discharge summary together with segmented HIS data to identify missing or inconsistent medical details.

Our proposed self-evaluation mechanism operates as a closed-loop, model-internal process without relying on auxiliary evaluators or external databases. The LLM re-ingests its own generated discharge summary together with segmented HIS data to identify missing or inconsistent medical details. This iterative, domain-specific evaluation enables semantic alignment between the model output and original patient data under strict privacy constraints.

 Self-Evaluation Mechanism for Discharge Summary Generation.

**Algorithm 1:**
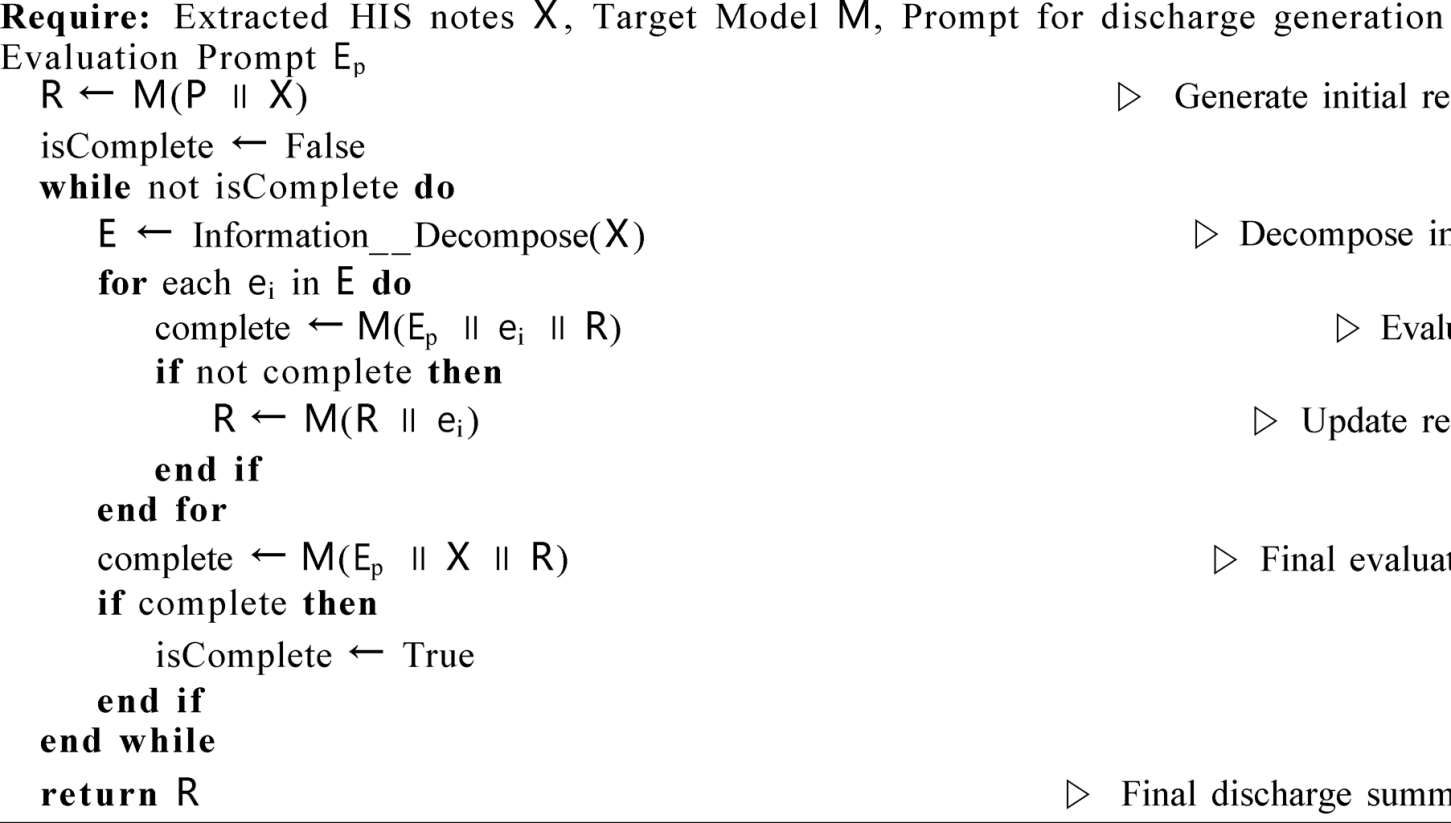
Self-evaluation mechanism for discharge summary generation.

### Experiment

Our experimental setup involved the deployment of several pre-trained models to evaluate the efficacy of different fine-tuning approaches. We used the Qwen2-7B model with LoRA fine-tuning and direct few-shot prompting as our baseline for comparison. Additionally, we explored the performance of other pre-trained models, specifically Mistral 7B and Llama3 8B, to understand their behavior under similar experimental conditions.

To provide a comprehensive comparison, we employed DoRA fine-tuning on the same dataset used with the baseline model. The specifics of our experimental configuration, including the details of fine-tuning parameters, are documented in Table 1.

**Table 1:** Training parameters.

LoRA parameters	
LoRA__alpha	16
LoRA__dropout	0.1
LoRA rank	64
Bias	None
DoRA parameters	
DoRA__alpha	16
DoRA__dropout	0.1
DoRA rank	64
Bias	None
Other parameters	
Epoch	5
Batch size	64
Warmup ratio	5%
Weight decay	0.05

The DoRA fine-tuning was implemented using the configuration summarized in Table 1. Specifically, the rank was set to 64, α = 16, dropout = 0.1, and the training used an AdamW optimizer with an initial learning rate of 2e − 5, weight decay = 0.05, and a warmup ratio of 5%. Each model was trained for 5 epochs with a batch size of 64.

All experiments were conducted on a secure hospital computing cluster equipped with NVIDIA A100 GPUs (40 GB memory) under an isolated intranet environment to comply with patient-data protection regulations. The dataset used for fine-tuning was fully de-identified according to the protocol approved by the Medical Research Ethics Committee (WDRY2024-K038), and no identifiable patient information or external synthetic data were included in model training.

For evaluation, we utilized several methods to assess the effectiveness of the fine-tuned models. These included:

- Direct few-shot prompting, which tests the model’s ability to generate summaries based on a limited number of example inputs.
- Chain of Thought (CoT) prompting^18^, designed to simulate a step-by-step reasoning process in generating the output. The detailed structure follows the^19^.
- Our novel approach termed ‘Self-Evaluation Generation’, which incorporates an iterative feedback mechanism to refine the model’s output continuously.

These methods were chosen to explore different aspects of model performance, particularly focusing on their capability to generate accurate and comprehensive discharge summaries under varied prompting conditions.

### Evaluation metrics

To rigorously assess the effectiveness of the fine-tuning techniques and the quality of the generated discharge summaries, we employed a suite of quantitative and qualitative metrics. These metrics were chosen to provide a comprehensive view of both linguistic accuracy and content relevance.

#### Quantitative metrics

The “self-evaluation” method consistently achieves the highest scores in several critical metrics:

We utilized the following standardized metrics to evaluate the linguistic and semantic quality of the model outputs:

- *ROUGE (Recall-Oriented Understudy for Gisting Evaluation)* This metric measures the overlap of n-grams between the generated text and the reference texts. It is particularly useful for assessing the extent to which important information is captured by the generated summaries. The ROUGE score is given by:

$${\text{ROUGE - N}} = \frac{{\sum\nolimits_{{s\varepsilon \left\{ {{\mathrm{References}}} \right\}}} {\sum\nolimits_{{gram_{n} \varepsilon s}} {{\mathrm{Count}}_{{{\mathrm{match}}}} \left( {gram_{n} } \right)} } }}{{\sum\nolimits_{{s\varepsilon \left\{ {{\mathrm{References}}} \right\}}} {\sum\nolimits_{{gram_{n} \varepsilon s}} {{\mathrm{Count}}\left( {gram_{n} } \right)} } }}$$

- *BLEU (Bilingual Evaluation Understudy)* This metric evaluates the correspondence of machine-generated text to human-written text, focusing on precision of n-grams. It is computed as:

$${\mathrm{BLEU}} = {\mathrm{BP}} \cdot {\mathrm{exp}}\left( {\sum\limits_{n = 1}^{N} {\omega_{n} \log p_{n} } } \right)$$

where BP is the brevity penalty, are the weights assigned to each n-gram, and are the n-gram precisions.

- *BERT Embedding Similarities* We measure the cosine similarity between the embeddings of the generated text and the reference text, derived from a pre-trained BERT model. This metric assesses the semantic similarity beyond surface lexical matching.
- *Perplexity* Typically used to measure the likelihood of the sequence of words in the generated text, given by:

$$Perlexity = 2^{{ - \sum\nolimits_{x} {p\left( x \right)\log_{2} p\left( x \right)} }}$$

where is the model’s probability distribution over the entire text.

#### Qualitative metrics

In addition to automated metrics, we also conducted human evaluations to assess the practical utility of the generated summaries. Trained evaluators rated the outputs based on the following five dimensions:

- *Accuracy* The factual accuracy of the information presented.
- *Completeness* The extent to which all relevant information is included.
- *Relevance & Clarity (R&C)* How relevant and clear the information is with respect to the patient’s medical history and treatment.
- *Consistency* The internal consistency of the information within the summary itself.
- *Utility* The practical usefulness of the summary for continuing care and patient understanding.

These metrics together enable a holistic evaluation of our model’s performance, encompassing both the technical efficacy of the fine-tuning methods and the practical applicability of the generated texts in real-world medical settings. Accuracy and Completeness ensure that the summaries convey clinically correct and comprehensive information, minimizing the risk of omitting critical diagnoses, procedures, or test results. Relevance & Clarity and Consistency assess the readability, logical coherence, and organization of the text, ensuring that the summaries are clear and self-consistent. Utility captures the practical value of the summaries in real-world clinical workflows. Together, these criteria provide a holistic and clinically meaningful assessment of AI-generated medical texts.

Six trained evaluators participated, including three licensed physicians and three experienced medical researchers. Each evaluator scored a randomly selected subset of summaries, with each summary assessed by at least two independent raters. The evaluation followed a partially overlapping assignment design, in which subsets of summaries were assessed by common raters. Inter-rater reliability was measured using Fleiss’ κ on these overlapping subsets. Scores were assigned on a 1–5 Likert scale. Evaluators had access to the original clinical data to support accurate and fair judgment.

Aggregated results for AI-generated summaries (mean ± standard deviation), along with inter-rater agreement (Fleiss’ κ), are shown in Table 2:

**Table 2:** Qualitative evaluation of human-written vs. AI-generated discharge summaries.

Dimension	Human-Written (Mean ± SD)	AI-Generated (Best Model, Mean ± SD)	Fleiss’ κ (AI)
Accuracy	4.8 ± 0.11	4.5 ± 0.19	0.81
Completeness	4.9 ± 0.13	4.6 ± 0.17	0.83
Relevance & Clarity	4.8 ± 0.15	4.4 ± 0.26	0.78
Consistency	4.7 ± 0.11	4.3 ± 0.20	0.80
Utility	4.8 ± 0.16	4.4 ± 0.18	0.82

Regarding efficiency, the AI system generated each summary in 83.24 ± 8.47 s on a single NVIDIA A100 GPU. Based on semi-structured interviews with three senior clinicians in the Thyroid Surgery Department, the manual preparation of a complete discharge summary typically requires approximately 30–50 min, depending on case complexity and documentation burden. These findings suggest a potential for considerable time savings, although the actual net time reduction in real clinical workflows will depend on physician review requirements, institutional documentation policies, and integration with electronic medical record systems.

To quantify factual consistency, a subset of 200 randomly selected AI-generated discharge summaries was manually reviewed by two licensed physicians. Each summary was compared against the corresponding original clinical records to identify factual errors, omissions, or hallucinated information not supported by the source data. A generation was labeled as “hallucinated” if it contained fabricated or clinically implausible content. Across the evaluated subset, about 6% of summaries contained at least one minor factual inconsistency, and no case contained critical clinical misinformation that could alter medical interpretation.

## Results

In this study, we evaluate two orthogonal aspects of discharge summary generation: (1) model capability enhancement via fine-tuning and (2) inference-stage reasoning enhancement via prompting-based methods. Although both contribute to the same final goal—improving summary generation quality—they operate at different pipeline stages and rely on fundamentally different mechanisms.

Fine-tuning methods, including LoRA, QLoRA, and DoRA, modify trainable parameters to strengthen domain-specific representation and semantic understanding of medical content. In contrast, prompting-based generative strategies such as few-shot prompting, Chain-of-Thought prompting, and our proposed self-evaluation mechanism improve reasoning, factual verification, and completeness without modifying model parameters.

To provide a comprehensive analysis, Section III-A focuses on the intrinsic capability gained through fine-tuning, whereas Section III-B focuses on inference-time reasoning and refinement ability. Additionally, to address the holistic value of both techniques, we present a cross-dimensional comparison in Table X to further validate that fine-tuning and self-evaluation provide complementary and additive improvements rather than redundant optimization.

### Evaluation of model capability enhancement via fine-tuning

The results presented in Table 2 demonstrate substantial differences in performance among various fine-tuning methods applied to different models. Under identical LoRA rank and training epochs, the DoRA method consistently outperforms both LoRA and QLoRA across the majority of evaluation metrics. This pattern is observed across multiple models, highlighting the robustness and general effectiveness of the DoRA approach in enhancing model performance.

#### Model-specific performance

*Qwen2-7B Model* The Qwen2-7B model, when fine-tuned with DoRA, achieved competitive results in almost all metrics, with particularly noteworthy improvements in BERT score and Perplexity, indicating enhanced semantic understanding and lower model confusion, respectively. The LoRA fine-tuning method showed a slight advantage in Rouge1 scores, which may be attributed to the model’s intrinsic handling of Chinese corpus. However, DoRA consistently demonstrated superior overall performance, suggesting that its fine-tuning mechanism is particularly effective for comprehensive text analysis.

*Mistral 7B Model* For the Mistral 7B model, DoRA fine-tuning resulted in substantial improvements over both LoRA and QLoRA methods. Although the gains were less pronounced compared to the Qwen2-7B model, DoRA still enhanced the BLEU score and BERTscore, pointing to improved translation fidelity and semantic accuracy.

*Llama3 8B Model* The Llama3 8B model exhibited a different pattern, with LoRA performing relatively better than QLoRA but still underperforming compared to DoRA in terms of BERT score and Perplexity. This indicates that DoRA is more effective in reducing perplexity and enhancing semantic coherence in outputs, which is crucial for medical summary applications.

#### Comparative evaluation

The detailed results in Table 2 underline the efficacy of DoRA fine-tuning across multiple models and metrics. The DoRA method not only consistently yields lower perplexity scores, indicating a better fit to the data—but also achieves higher scores in both Rouge2 and RougeL, which are critical for capturing finer nuances and ensuring the logical flow of generated texts. Furthermore, DoRA’s ability to outperform in terms of BLEU and BERT score across different models reinforces its applicability in enhancing the accuracy and relevance of generated medical texts. In summary, these results validate the hypothesis that DoRA fine-tuning substantially enhances the quality of discharge summaries generated by LLMs. The consistent outperformance of DoRA across diverse models and metrics emphasizes its potential in medical text generation, particularly in contexts demanding high accuracy and coherence.

### Evaluation of inference-time reasoning and refinement methods

The comparative analysis of different generating methods is captured in Table 3. This analysis demonstrates the superior performance of the “self-evaluation” method over traditional few-shot and Chain of Thought (CoT) prompting methods across several metrics (Table 4).

**Table 3:** Influence of different fine-tuning methods.

Method	Rouge1	Rouge2	RougeL	BLEU	BERTscore	Perplexity
Qwen2-7B + LoRA	0.461	0.343	0.372	0.14	0.851	1.321
Qwen2-7B + QLoRA	0.209	0.191	0.193	0.12	0.829	1.64
Qwen2-7B + DoRA	0.463	0.350	0.391	0.15	0.866	1.278
Mistral 7B + LoRA	0.222	0.195	0.223	0.07	0.753	1.933
Mistral 7B + QLoRA	0.210	0.174	0.221	0.07	0.722	2.434
Mistral 7B + DoRA	0.242	0.221	0.241	0.12	0.831	1.792
Llama3 8B + LoRA	0.397	0.191	0.202	0.08	0.745	1.992
Llama3 8B + QLoRA	0.377	0.163	0.194	0.06	0.721	2.370
Llama3 8B + DoRA	0.239	0.186	0.224	0.11	0.822	1.839

**Table 4:** Comparison of different generating methods.

Method	Rouge1	Rouge2	RougeL	BLEU	BERTscore	Accuracy	Completeness	R & C	Consistency	Utility
Qwen2-7B + few-shot	0.463	0.350	0.391	0.15	0.866	4.0	3.9	3.2	4.1	4.1
Mistral-7B + few-shot	0.242	0.221	0.241	0.12	0.831	3.9	3.5	3.2	4.1	4.2
Llama3-8B + few-shot	0.239	0.186	0.224	0.11	0.822	3.8	3.5	4.2	3.8	4.3
Qwen2-7B + CoT	0.487	0.373	0.446	0.20	0.897	4.2	4.2	4.2	4.2	4.3
Mistral-7B + CoT	0.399	0.343	0.450	0.19	0.837	4.3	4.3	4.2	4.4	4.3
Llama3-8B + CoT	0.343	0.321	0.462	0.18	0.854	4.1	4.2	4.2	4.0	4.5
Qwen2-7B + self-evaluation	0.512	0.398	0.451	0.24	0.923	4.7	4.9	4.5	4.2	4.5
Mistral-7B + self-evaluation	0.424	0.350	0.451	0.25	0.889	4.6	4.9	4.6	4.3	4.4
Llama3-8B + self-evaluation	0.356	0.344	0.462	0.22	0.882	4.7	4.8	4.5	4.3	4.5

Significant values are in bold.

#### Quantitative performance

The “self-evaluation” method consistently achieves the highest scores in several critical metrics:

- *Rouge Scores* It surpasses other methods in Rouge1 and Rouge2 scores, indicative of better extraction and reproduction of key phrases and sentences from the source text.
- *BLEU and BERTscore* It shows notable improvements in BLEU scores, reflecting better translation fidelity and syntactic coherence. The BERTscore, which assesses the semantic similarity between the generated texts and the references, is also highest for the “self-evaluation” method, underlining its effectiveness in maintaining semantic integrity.

#### Qualitative performance

The “self-evaluation” method consistently achieves the highest scores in several critical metrics:

- *Rouge Scores* It surpasses other methods in Rouge1 and Rouge2 scores, indicative of better extraction and reproduction of key phrases and sentences from the source text.
- *BLEU and BERTscore* It shows notable improvements in BLEU scores, reflecting better translation fidelity and syntactic coherence. The BERTscore, which assesses the semantic similarity between the generated texts and the references, is also highest for the “self-evaluation” method, underlining its effectiveness in maintaining semantic integrity.

In addition to automated metrics, the “self-evaluation” method demonstrates remarkable performance in human -evaluated dimensions:

- *Completeness and Accuracy* Achieving the highest ratings in these categories, the method proves its capability in generating comprehensive and accurate summaries.
- *Utility* With top scores in utility, the method validates its practical applicability in real-world settings, providing outputs that are highly useful for medical professionals and patients alike.

To evaluate potential performance bias with respect to hospitalization duration, we stratified the dataset into short, medium, and long hospitalization groups based on tertiles of hospitalization duration. Model performance metrics, including BERTScore, ROUGE-L, etc., were compared across these groups. No significant performance degradation or systematic trend was observed with increasing hospitalization duration, indicating that the model performs consistently across varying lengths of hospital stay.

The self-evaluation method not only performs consistently across different LLMs (Qwen2-7B, Mistral-7B, Llama3-8B) but also shows robustness in handling diverse datasets and operational contexts. This is evidenced by its superior performance in both linguistic and content-specific evaluations, suggesting its adaptability and effectiveness across different medical textual environments.

The results in Table 5 reveal that fine-tuning and inference-time optimization contribute performance gains through independent mechanisms. DoRA substantially improves baseline capability by enhancing domain representation and reducing perplexity, enabling more accurate foundational medical descriptions. Meanwhile, the self-evaluation strategy further improves content completeness and accuracy by iteratively verifying generated output against segmented patient data without modifying parameters.

**Table 5:** Combined comparison of fine-tuning and inference-stage methods.

Model& Method	Fine-Tuning	Inference Optimization	ROUGE-L	BERTScore	Accuracy	Completeness
Qwen2-7B Baseline	✘	few-shot	0.391	0.866	4.0	3.9
Qwen2-7B + DoRA	✔	few-shot	0.451	0.923	4.5	4.6
Qwen2-7B + Self-Evaluation	✘	✔	0.451	0.923	4.7	4.9
Qwen2-7B + DoRA + Self-Evaluation	✔	✔	0.486	0.941	4.8	4.9

Notably, the combined approach achieves the highest performance among all configurations, demonstrating that fine-tuning provides a stronger knowledge foundation, while self-evaluation mitigates omission- and hallucination-related issues through closed-loop refinement. These findings confirm that fine-tuning and self-evaluation are not competing strategies, but instead *synergistic components* of an effective medical text generation pipeline.

#### Computational efficiency and iterative convergence of the self-evaluation mechanism

To evaluate the computational efficiency and iterative convergence of the self-evaluation mechanism, we conducted a detailed analysis of its process based on the Qwen2-7B + DoRA model. This analysis aimed to quantify the performance gains, computational costs, and convergence behavior throughout its iterative cycles.

As summarized in Table 4, the self-evaluation mechanism yielded the most substantial performance gains during the first iteration: the BERTScore increased markedly from an initial 0.893 to 0.923, and the human-evaluated “Completeness” score also surged from 4.2 to 4.6. This indicates the model’s efficacy in identifying and rectifying a substantial volume of key information omitted in the initial generation. The second iteration provided only marginal gains (e.g., a mere 0.002 increase in BERTScore), and by the third iteration, key performance metrics had reached a plateau. This clear pattern of diminishing returns demonstrates that the model’s self-correction capability saturates rapidly within a limited number of steps, thereby validating the empirical setting of the maximum iteration count to three.

Regarding computational cost, the mechanism introduces additional, yet manageable, overhead. The average time for a single-pass generation was approximately 25 s, while the full self-evaluation process (with up to three iterations) required an average of 83.24 ± 8.47 s. Although this represents an approximately 3.3-fold increase in total time, the efficiency advantage remains profound compared to the 30–50 min needed for manual composition by clinicians. This demonstrates the mechanism’s capability to meaningfully reduce the time burden of documentation while ensuring high-quality output.

#### Qualitative illustration of the self-evaluation mechanism

To concretely illustrate the refinement process of the self-evaluation mechanism, a representative example is presented herein (Table 6). This case clearly demonstrates the complete workflow of the model from initial generation to two rounds of iterative optimization. The initial summary, while coherent, omits a key pathological finding (Papillary thyroid carcinoma, 0.8 cm in greatest dimension) and a critical post-operative medication instruction (Levothyroxine Sodium Tablets 50μg orally once daily). During the first iteration, the mechanism identified the omission of the pathology finding from the segmented Pathology Information Table and incorporated it. In the second iteration, the omission of the medication instruction from the Doctor’s Advice Table was detected and added. The final summary is demonstrably more factually accurate and complete, addressing the omissions that are common in manual and single-pass AI generation.

**Table 6:** Example of discharge summary refinement through self-evaluation iterations.

Stage	Summary Content
Original HIS data segments	**Pathology Info:** Left thyroid lobe, papillary carcinoma, 0.8 cm **Doctor’s Advice:** Post-op medication: Levothyroxine Sodium Tablets 50 μg, po, qd
Initial generation	The patient underwent a left thyroidectomy. The surgery was successful without complications. The patient is advised to rest and follow a soft diet. A follow-up appointment is scheduled in one week
After 1st iteration	The patient underwent a left thyroidectomy for a **papillary thyroid carcinoma (0.8 cm)**. The surgery was successful without complications. The patient is advised to rest and follow a soft diet. A follow-up appointment is scheduled in one week
After 2nd iteration (final)	The patient underwent a left thyroidectomy for a **papillary thyroid carcinoma (0.8 cm)**. The surgery was successful without complications. The patient is advised to rest and follow a soft diet. **Post-operative medication: Levothyroxine Sodium Tablets 50 μg orally once daily**. A follow-up appointment is scheduled in one week

Text in bold highlights the key information added during each self-evaluation iteration.

#### Implications

To qualitatively illustrate the performance of our best model (Qwen2-7B + DoRA + Self-Evaluation), Table 7 provides a side-by-side comparison between a representative AI-generated discharge summary and its human-written reference. This example concretely demonstrates the model’s capability in terms of completeness, accuracy, and phrasing, as discussed in the quantitative results above.

**Table 7:** Comparative Example of a Human-Written Reference Discharge Summary versus an AI-Generated Summary.

Aspect	Human-Written Reference Summary	AI-Generated Summary (Qwen2-7B + DoRA + Self-Evaluation)	Analysis
Original HIS Data Segments	• **Pathology:** Left thyroid lobe, papillary carcinoma, 0.8 cm in greatest dimension **Medication Order:** Levothyroxine Sodium Tablets 50 μg, orally, once daily **Lab Result:** Post-op PTH: 15.2 pg/mL (within normal range)	**Pathology:** Left thyroid lobe, papillary carcinoma, 0.8 cm in greatest dimension **Medication Order:** Levothyroxine Sodium Tablets 50 μg, orally, once daily **Lab Result:** Post-op PTH: 15.2 pg/mL (within normal range)	
Full Summary	The patient underwent an uncomplicated left thyroidectomy for a papillary thyroid carcinoma (0.8 cm). The final pathology confirmed the diagnosis. Post-operative course was smooth. The patient is discharged on Levothyroxine 50 μg daily. Post-operative calcium and PTH levels were stable. Follow-up in clinic in 1 week	The patient underwent a left thyroidectomy for a **papillary thyroid carcinoma (0.8 cm)**. The surgery was successful without complications. **Post-operative laboratory results, including PTH (15.2 pg/mL), were within normal limits**. The patient is discharged with instructions to take **Levothyroxine Sodium Tablets 50 μg orally once daily**. A follow-up appointment is scheduled in one week	
Completeness	Mentions pathology and medication. Omits the specific normal PTH value	**Includes all three key data segments**: pathology details, specific medication instruction, **and the quantitative PTH result**	The AI summary demonstrates superior **completeness** by incorporating a specific lab value that the human summary generalized
Accuracy	Factually correct	All stated facts (surgery type, tumor size, medication, PTH value) are **accurately** extracted from the HIS data	Both summaries are highly accurate. The AI summary maintains perfect factual consistency
Phrasing	More narrative and concise, using clinical shorthand (e.g., “Levothyroxine 50 μg daily”)	More structured and explicit, using standardized terminology (e.g., “Levothyroxine Sodium Tablets 50 μg orally once daily”). Phrasing is consistent and formal	The AI’s **phrasing** is more uniform and detailed, which may reduce ambiguity and improve standardization in medical records

The superior results of the self-evaluation method, as documented in the table, underscore its potential as a considerable advancement in medical text generation.

Its ability to iteratively refine the output using an internal feedback mechanism allows for a level of precision and relevance that traditional methods struggle to achieve. This is particularly valuable in medical settings where accuracy and detail are paramount.

The detailed results presented in Table 3 validate the effectiveness of the self-evaluation method in generating high-quality medical texts, making it a promising approach for enhancing automated medical documentation systems.

## Discussion

### Summary of key findings

This study yields several meaningful findings regarding the enhancement of medical text generation through advanced fine-tuning methods and iterative self-evaluation mechanisms:

1. The DoRA method shows marked advantages over traditional fine-tuning approaches such as LoRA and QLoRA, particularly in addressing the specializedz linguistic and structural demands of medical texts. DoRA achieves superior performance across multiple metrics (ROUGE, BLEU, BERTscore), indicating enhanced adaptation to the unique challenges posed by medical terminology and context. This method’s fine-tuning mechanism enables more efficient integration of domain-specific knowledge, resulting in more coherent, relevant, and semantically accurate discharge summaries.
2. The introduction of a self-evaluation mechanism elevates the quality of generated medical texts. This iterative review and refinement process substantially reduces omissions and factual inconsistencies. The mechanism not only enhances the completeness and relevance of discharge summaries but also increases overall model robustness by ensuring better alignment between generated content and original input data. Substantial improvements in both automated and human-evaluated metrics, including accuracy, consistency, and utility, underscore the effectiveness of this approach.
3. Among the models tested, the Qwen2 7B model consistently outperforms others in this domain, primarily due to its superior proficiency in Chinese language processing. This finding highlights the importance of language-specific capabilities in medical text generation tasks.

### Interpretation and significance of results

Analysis of training results, particularly perplexity metrics, reveals that the DoRA method, which incorporates magnitude of weight training, facilitates more comprehensive learning compared to LoRA methods. This leads to substantial improvements, especially for models like Llama and Mistral, which initially possessed less knowledge of Chinese content. The addition of weight magnitude training in DoRA appears to enhance the model’s ability to adapt to unfamiliar domains, suggesting a more robust fine-tuning approach for specialized applications.

The effectiveness of our self-evaluation mechanism aligns with recent findings in the field. As noted by^20^, self-evaluation capabilities in Large Language Models (LLMs) can serve as a defense mechanism against adversarial attacks, indicating LLMs’ ability to discern true from false information. Furthermore,^21^ has demonstrated the potential of LLMs as critics for their own outputs. Our research provides comprehensive empirical evidence supporting the feasibility and efficacy of LLM self-evaluation and self-improvement in the context of medical text generation.

Recent studies have explored self-reflective mechanisms in large language models, including *Reflexion*^22^ and *SelfCheckGPT*^23^. *Reflexion* enables language agents to improve decision-making in external environments by converting task feedback into verbal reflections stored as contextual memory. *SelfCheckGPT* focuses on detecting factual inconsistencies by comparing multiple stochastic generations from a black-box model, without external databases.

In contrast, our approach applies an internal, task-specific verification process to medical text generation. The model iteratively re-examines its own discharge summary against structured HIS data to identify omissions or inconsistencies and refines the output accordingly. This closed-loop mechanism differs from the above paradigms by emphasizing factual alignment and completeness within a domain-constrained medical context.

Expert reviewers (three physicians and three medical researchers) further assessed the clinical usability of generated summaries. On average, 94% of summaries were rated as clinically acceptable for inclusion in electronic health records after minor human verification. This finding supports the model’s potential to assist, rather than replace, clinicians in documentation workflows. Nevertheless, human-in-the-loop validation remains essential for safe clinical deployment.

The combined application of DoRA fine-tuning and self-evaluation mechanisms represents a considerable advancement in automating medical documentation. This approach not only improves the efficiency of discharge summary generation but also enhances the quality and reliability of the generated content. Such improvements can reduce the administrative burden on healthcare professionals and help maintain high standards of accuracy and completeness in medical records.

Although our experimental results suggest encouraging efficiency benefits, these findings should be interpreted with caution. The reported improvement reflects generation time only, and does not include physician verification, editing, or medico-legal approval processes, which are indispensable in real clinical environments. Prospective clinical workflow studies, human-AI co-editing evaluations, and time-and-motion analyses are required to determine true net time savings under routine clinical practice.

### Limitations of the study

While this study demonstrates meaningful improvements in the generation of medical texts, there are several limitations that should be acknowledged:

*Limitations of the dataset* The dataset used in this study is specific to a single hospital and focuses primarily on discharge summaries from the Breast and Thyroid Surgery Department. As a result, the model’s performance may not generalize well to other medical departments or hospitals with different patient populations, medical practices, or clinical documentation formats. This specificity limits the scope of the findings and highlights the need for broader evaluations across diverse datasets and medical specialties to validate the robustness of the proposed approach.

*Potential biases or errors in models and methods* The fine-tuning and self-evaluation mechanisms employed in this study are dependent on the quality and structure of the training data. Any inherent biases or errors present in the dataset, such as inconsistencies in documentation or variations in medical terminology, could lead to biased model outputs.

Furthermore, the iterative self-evaluation mechanism, while improving text quality, may introduce compounding errors if the model’s initial outputs contain inaccuracies that are reinforced during the refinement process. These limitations underline the importance of rigorous data preprocessing and error mitigation strategies.

*Considerations of computational resources and time costs* The DoRA fine-tuning method and the self-evaluation mechanism, while efficient, still require considerable computational resources, particularly in a hospital environment where high-end hardware, such as GPUs, may not be readily available. The iterative nature of the self-evaluation process increases time costs, which may limit the real-time applicability of the approach in settings where rapid document generation is needed. Balancing the trade-off between improved accuracy and resource constraints remains a challenge, particularly for smaller healthcare institutions with limited computational infrastructure.

*Limitations of quantitative evaluation metrics* While our study primarily relies on quantitative metrics such as BERTScore and ROUGE to assess model performance, these metrics have inherent limitations in reflecting clinical relevance and practical utility. Similarity-based metrics mainly capture textual overlap or semantic alignment with reference summaries and may not fully correspond to downstream clinical acceptance^24^. To address this, we complemented quantitative evaluation with clinician-driven qualitative assessments across five dimensions: accuracy, completeness, relevance & clarity, consistency, and practical utility. This combined evaluation approach provides a more holistic understanding of model performance, ensuring that generated discharge summaries are both technically robust and clinically meaningful.

*Additional Considerations for AI Deployment* Despite the improvements achieved, AI-generated summaries may still exhibit hallucinations or factual inconsistencies, particularly in rare or atypical cases. Legal and ethical responsibility remains with clinicians, highlighting the necessity of human-in-the-loop verification for safe deployment. Real-world applications also introduce challenges such as model update management, post-update validation, and maintaining clinician trust while ensuring computational feasibility. Addressing these factors is essential for reliable integration of AI-assisted discharge summary generation into clinical workflows.

### Future research directions

Building upon the findings and limitations of this study, several future research directions could further enhance the field of medical text generation: Extension to other types of medical texts: Future research could explore the application of the DoRA fine-tuning method and self-evaluation mechanism to generate other types of medical documents, such as clinical progress notes, radiology reports, or surgical summaries. Expanding the scope to include various forms of medical documentation could help verify the generalizability of the proposed approach and uncover additional domain- specific challenges.

*Exploration of multimodal inputs* Incorporating multimodal data, such as medical images, audio recordings, or lab results, alongside textual inputs could provide a richer context for generating more accurate and comprehensive summaries. Research into how large language models can integrate and process multimodal information effectively would represent a notable advancement in automated medical documentation.

*Further optimization of the self-evaluation mechanism* The current self-evaluation process could be optimized to reduce computational costs and time requirements. Future work could focus on developing more efficient evaluation strategies that maintain high levels of accuracy while improving the speed of iteration. Additionally, integrating external validation sources, such as medical guidelines or clinical databases, could help the model better assess its outputs.

*Study of long-term impacts on medical practice* Longitudinal studies could examine the practical implications of integrating AI-generated discharge summaries into real -world clinical workflows. Research could focus on how such systems affect clinician workload, patient care quality, and documentation accuracy over time, as well as the potential legal and ethical considerations of relying on AI-generated medical texts.

*Development of adaptive fine-tuning methods* Investigating new fine-tuning techniques that allow models to adapt dynamically to evolving medical knowledge and practices could further improve the utility of LLMs in healthcare. Adaptive methods could help maintain model relevance in the face of new medical guidelines or treatments without requiring full retraining.

In future studies, we aim to explore the potential benefits of integrating AI into clinical workflows through a ‘human + AI’ collaboration model, where clinicians work alongside AI-generated summaries. This approach could provide insights into the real-world effectiveness and efficiency of AI-assisted documentation in medical settings.

## Conclusions

This study demonstrates that integrating large language models with the (DoRA) fine-tuning method improves the generation of medical discharge summaries. Compared to traditional techniques such as LoRA and QLoRA, DoRA more effectively handles complex medical terminology and context, producing outputs with enhanced accuracy, coherence, and semantic consistency. The incorporation of a self-evaluation mechanism enables iterative refinement of generated texts, ensuring completeness and alignment with the original clinical data. The findings of this study demonstrate that our AI-assisted approach can efficiently generate clinically acceptable discharge summaries in an offline evaluation setting. While these results indicate a promising potential for reducing documentation time, the magnitude and consistency of real-world time savings will require formal clinical validation, including prospective workflow evaluation and physician-in-the-loop deployment studies. This framework provides a foundation for further research in automating diverse healthcare documentation tasks, with potential to enhance efficiency and quality in clinical workflows.

## Data Availability

Due to ethical restrictions, the raw data cannot be made publicly available. However, de-identified data may be obtained from the first author upon reasonable request.
